# The Effect of Orthodontic Forces on Tooth Response to Electric Pulp Test

**DOI:** 10.7508/iej.2015.04.007

**Published:** 2015

**Authors:** Jalil Modaresi, Hosein Aghili, Omid Dianat, Farnaz Younessian, Faranak Mahjour

**Affiliations:** a *Department of Endodontics, Dental School, Social Determinants of Oral Health Research Center, Shahid Sadoughi University of Medical Sciences, Yazd, Iran;*; b * Department of Orthodontics, Dental School, Shahid Sadoughi University of Medical Sciences, Yazd, Iran; *; c * Iranian Center for Endodontic Research, Research Institute for Dental Sciences, Department of Endodontics, Dental School, Shahid Beheshti University of Medical Sciences, Tehran, Iran; *; d *Dentofacial Deformities Research Center, Research Institute for Dental Sciences, Dental School, Shahid Beheshti University of Medical Sciences, Tehran, Iran; *; e *Department of Molecular and Cell Biology, Henry M. Goldman, Medical School, Boston University, Boston, MA, USA*

**Keywords:** Electric Pulp Test, Orthodontic Treatment, Pulp Vitality

## Abstract

**Introduction:**

The current study investigated the pulp response to electric pulp testing (EPT), before, upon initiation and one month after the start of orthodontic tooth movement.

**Methods and Materials:**

A total of 402 anterior teeth from 39 patients (mean age of 16.8±2.7 years) were examined in this non-controlled prospective study. The aligning forces were administered using initial NiTi archwires ligated on fixed appliances by using the MBT straight wire technique. The electrical stimulation was provided by the EPT. The EPT readings were recorded at three time points: before bonding (EPT_0_), immediately upon initiation (EPT_1_) and 1 month post-treatment (EPT_2_). The data were statistically analyzed by the ANOVA and Bonferroni tests (*P*<0.05).

**Results:**

Prior to bonding of the orthodontic brackets, the mean EPT value for all the experimental teeth was 3.42 EPT units. Upon initiation, the mean value of EPT_1_ for each tooth increased to 7.62 units. One month later, the mean EPT_2_ values dropped to 6.27 units. At this time point, 64 teeth (16%) of the experimental teeth failed to respond. The differences among EPT values at different time points were significant. There was no association between the EPT values and the location or the type of teeth.

**Conclusion:**

The physiological changes in the pulp affect the nerve fibers in the early stages of the orthodontic force application. As a result, thresholds to electrical stimulation would increase and the EPT may not initiate a response. Therefore results obtained by electrical pulp testing should be interpreted accordingly.

## Introduction

The impact of orthodontic forces on the dental pulp tissue has become a matter of interest [1, 2]. Several studies have evaluated the impact of orthodontic forces on the dental pulp. However, the reported results in the literature are inconsistent and inconclusive, mostly due to the methodological limitations [3]. Some studies have reported short term effects such as changes in tissue respiration [4, 5], and others have reported long lasting consequences such as necrosis [6, 7].

Histological examination, is neither practical nor feasible in clinical situation. Therefore, application of pulp testing methods is suggested to provide additional diagnostic information [8]. Different pulp tests have been proposed and examined aiming at assisting the diagnosis and treatment planning for the clinician [9]. Laser Doppler flow cytometry (LDF) is a proposed method for evaluating the blood flow in the dental pulp by investigating the vascular supply to the pulp [2, 10]. This method contains some limitations such as highly expensive equipment, high technique sensitivity, long experiment period and possibility of false readings from the periodontium [8].

Another alternative method is electrical pulp testing (EPT); a simple non-invasive test that provides the clinician with the electrical responsiveness of the pulp. This method provokes a group of fast-acting low-threshold *Aδ* fibers, present within the pulp. EPT can be used as a sensitivity test and would provide the clinician with qualitative sensory manifestations [8]. Although the results of this test might be accompanied with errors, prior knowledge about the situations in which the results might get aberrant, can reduce the potential errors [11]. EPT only provides information on the status of pulpal nerves, and does not directly determine the vitality (vascularity) of pulp [11]. However, a positive response to EPT is generally interpreted as the pulp vitality. Based on the duration of the response as well as the history of the patient, the clinician can judge whether the pulp is healthy or inflamed [10]. Changes in the physiology of the pulp might have some effects on pulpal nerves, especially *Aδ* and *Aβ* fibers, which can cause alterations to the EPT results [12, 13].

A number of studies have examined the effect of orthodontic force on pulp responses to EPT. Some studies have reported an increased level of sensitivity to EPT in teeth undergoing orthodontic forces [14]. On the other hand, some of them reported decreased level of sensitivity [15-18]. 

Since little and conflicting results have been reported regarding the response of orthodontically treated teeth to EPT [6, 11, 17, 19], the purpose of the current non-controlled prospective study was to investigate the response of teeth to EPT before (EPT_0_), immediately after (EPT_1_), and one month subsequent to the initiation of orthodontic force (EPT_2_) with pre-adjusted MBT bracket system.

## Materials and Methods

A total of 39 patients (18 males and 21 females) providing 402 anterior maxillary and mandibular teeth participated as experimental group in this non-controlled prospective study. The mean age was 16.8±2.7 years (with a range of 13-22 years). All cases in the study group had class I malocclusion with moderate crowding (irregularity index was between 5 and 10). Other inclusion criteria were: the need for non-extraction fixed orthodontic treatment, no systemic diseases, no consumption of any medications, healthy periodontium (probing depths not exceeding 3 mm, no bone loss as determined by radiographs) and sound dentition (no carious lesions, restorations or history of trauma, absence of missing teeth and extraction, closed apex), no endodontically treated teeth, positive initial EPT responses before orthodontic treatment and no previous removable orthodontic appliances. Written informed consent was obtained from the patients and their parents for those younger than 18. The study protocol was approved by the Ethics Committee of Shahid Sadoughi Medical University.

The 0.022"×0.028" slot straight wire system brackets were used and 0.016 round NiTi wires were used in all cases. The aligning forces were achieved using Sentalloy NiTi archwires (NiTi, GAC International Inc, Bohemia, NY, USA) that were ligated on the maxillary and mandibular fixed appliances on the permanently erupted teeth based on the MBT Straight Wire technique. NiTi archwires could exert constant physiological force for tooth movement irrelevant to the amount of their deflection [15]. 

Electrical stimulation was provided by the EPT device (Parkell, Farmingdale, NY, US) with toothpaste used as the conduction medium. Examination procedures were performed by the same operator and same EPT unit. The electrical stimuli was applied to the experimental teeth (maxillary and mandibular central incisors, lateral incisors and canines). Every tooth was isolated with cotton rolls and was dried thoroughly before EPT evaluation. To avoid contact with the orthodontic brackets, and to minimize the risk of false-positive responses elicited by inadvertent stimulation of the periodontal nerve fibers, or stimulation of adjacent teeth, the testing site was confined to sound enamel on the midpoint incisal edge of each tooth. The probe did not touch orthodontic brackets. Testing of each tooth started upon contact of the electrode tip on the tooth surface and terminated when the subjects raised their hands to show feeling of the first sensation (heat or tingling). The EPT had an analog display with a reading from 0 to 10. During testing, current flow was increased slowly from the initial zero current state by adjusting the variable voltage control. To minimize the procedural errors, a double determination method was used. Testing was repeated after a 3-min interval to reduce the subjective fatigue and to minimize the possibility of nerve accommodation. Examination procedures were performed by the same operator. The kappa values for the repeated recordings of the pulp response in the study groups varied between 0.8 and 1.0.

The numerical values on the EPT display were recorded at three treatment points: prior to bonding of orthodontic brackets (EPT_0_); immediately (5 min) after bonding and ligation of initial archwires (EPT_1_) and 4 weeks subsequent to initiation of tooth movement after archwire removal (EPT_2_). Teeth that failed to respond to electric testing were recorded as a reading of 10 EPT units. The following clinical and radiologic criteria were used to define pulp necrosis: loss of pulpal sensitivity and color changes in the crown [16].

The repeated measures analysis of variance (ANOVA) was used to assess the effect of time, location of the experimental tooth (upper or lower jaw) and the type of the experimental tooth on the EPT values. Significant difference between the EPT values in different treatment intervals was statistically analyzed by the Bonferroni test. The level of statistical significance was set at 0.05.

## Results

Within the first two time points (EPT_0 _and EPT_1_) all teeth responded positively to the EPT. After four weeks, 64 teeth (16% of the total) failed to respond to EPT. No tooth showed the signs of pulp necrosis during the experimental period.

Summaries of results recorded for the experimental teeth are shown in Table 1. Prior to bonding of orthodontic brackets (EPT_0_) the mean values for all experimental teeth was 3.42 EPT units (95% confidence intervals from 3.26 to 3.60). After bonding of the orthodontic brackets and ligation of the initial archwire, the mean threshold (EPT_1_) of each tooth increased to 7.62 EPT units (95% CI from 7.30 to 7.94; *P*<0.001) which was indicative of a significant decrease in the sensitivity to the test compared to EPT_0 _recordings. As treatment progressed, the mean EPT_2_ readings significantly decreased for each tooth and dropped to 6.27 units (95% CI from 6.05 to 6.49; *P*<0.001). However, the EPT_2_ mean threshold was still ~2.8 units (95% CI from 2.6 to 3.0; *P*<0.001) higher than the EPT_1_ values. No relation was observed between the pulp tester values and the location of experimental tooth (upper or lower jaw) (*P*=0.42) and type of the experimental tooth (*P*=0.13).

## Discussion

In the present study, the response of teeth to EPT before and during orthodontic treatment, was evaluated. Minutes after orthodontic load, increased EPT values were recorded, while one month later the threshold decreased but remained higher than the pre-treatment records.

The EPT was used in this study because it is noninvasive, simple and clinically popular. The tip of the probe was applied on sound enamel on the midpoint incisal edge of each tooth. This area is the most effective site for EPT because of its close proximity to the highly innervated pulp horns and because it provides a readily reproducible position for subsequent visits [12]. This placement also enabled avoiding contact with orthodontic bands and brackets. Attempts have been made to quantify the pulp response to EPT in previous studies [20-23]. The device has a numeric scale, and these numbers have been used to register changes in test responses. 

We used a voltage measuring pulp tester. Since electrical resistance may change according to environmental or biological conditions, a labial electrode was used in each subject to avoid the resistance changes.

**Table 1:** The pulp tester values for experimental teeth at three time points [before (EPT_0_), immediately after (EPT_1_) and one month after (EPT_2_) the initiation of treatment] (*P*<0.001)

**Time **	**Mean (SD)**	***P-value***
**EPT**_0_	3.42 (0.08)	0.008
**EPT**_1_	7.62 (0.15)	
**EPT**_2_	6.27 (0.10)	

Results of this study showed that almost immediately after initiation of orthodontic load, a significant increase in response threshold was observed in the experimental teeth. These results are consistent with the results reported by Cave *et al.* [21] and Burnside *et al.* [16] who stated this increase could also persist for up to 9 months after treatment. This could be stemming from immediate pressure or tension on apical nerve fibers. Previous investigations have shown that orthodontic force has an immediate effect upon pulp vascularity leading to hypoxia of the pulpal tissues [11]. It is suggested that these changes in the blood flow are temporary and return to normal within a few days. As a result, it can be hypothesized that changes in tissue respiration and possibly hypoxia that occur during orthodontic treatment may affect the pulp tissue and can alter the integrity of the *Aδ* and *Aβ* fibers, leading to an increase in the modified pulpal neural response [11].

In this study, we found no correlation between the pulp tester values and the location of experimental tooth (upper or lower jaw) or type of the experimental tooth. However, a previous study showed that different types of teeth respond to orthodontic forces differently [24]. Lateral incisors appear to tolerate a greater magnitude and duration of applied orthodontic pressure than central incisors, while canines appear to be the least affected teeth. This may be attributed to the smaller crown size and root surface area of the lateral incisors, and the larger root surface area of the canines which cause a difference in pressure applied on the root surface area [11]. 

Our results revealed that one month after application of orthodontic force, 16% of the total tested teeth failed to respond to EPT. However, none of them showed signs of pulp necrosis later. Our results are in agreement with the results of Cho *et al.* [25]. The explanation for this could be that there is a period after orthodontic treatment or trauma when the stimulus threshold to pulp testing might be so increased that a response would not be elicited [17]. Another study reported that 60 days after application of force, an elevation in response threshold occurred and the number of teeth failing to respond increased. This phenomenon is related to the time required to achieve initial leveling and alignment of the arches, and force levels on the teeth have been suggested to begin to dissipate after that [11]. On the other hand, some previous studies have reported pulp necrosis of non-traumatized teeth during orthodontic treatment [22, 23]. Possible explanations include anatomic variations of the apical foramen or the supplying vessels [24]. Further research should investigate whether pulpal sensitivity is associated with the common apical root resorption or perceived pain during orthodontic treatment. If there is any relationship between these items, EPT can preserve as a diagnostic tool during the orthodontic tooth movement. Further studies with non-metal brackets after archwire removal are also suggested.

## Conclusion

The physiological changes of the pulp affect the neural response in the early stages after application of orthodontic forces. Response thresholds to electrical stimulation are also increased and consequently the EPT may not initiate a response. This finding is not an indication of loss of pulp vitality because after a few weeks the response threshold decreases. Thus, dental practitioners should be cautious in their interpretation of electric vitality tests in patients undergoing orthodontic treatment.
